# Supplementary material for: Are carbon emissions trading and green financial instruments synergistic? -Comprehensive quantitative research based on content analysis
Source: PLoS One. 2024 Mar 7;19(3):e0298601. doi: 10.1371/journal.pone.0298601 (PMC10919601; doi:10.1371/journal.pone.0298601)
Supplement: S1 Data — (ZIP) [file pone.0298601.s001.zip › Data/地级市Z_F工作报告.zip!/╣π╢1⁄2/│▒╓▌╩╨/└┤╫╘╣1⁄2╓┌║┼í╛┬φ┐╦╩2╛▌═°í┐_2017.docx]

各位代表：

现在，我代表市人民政府向大会作工作报告，请予审议，并请市政协委员和其他列席人员提出意见。

过去五年工作回顾

过去五年，是我市经受住各种困难和考验，优化布局、科学发展，营造环境、夯实基础的五年。在省委、省政府和市委的正确领导下，在市人大及其常委会和市政协的监督支持下，市政府全面贯彻落实党的十八大和十八届中央历次全会精神，深入学习贯彻习近平总书记系列重要讲话精神，积极抢抓省振兴粤东西北发展机遇，围绕“一中心三片区”建设，坚持改革创新，统筹抓好稳增长、调结构、惠民生、防风险各项工作，经济社会实现平稳健康发展，“十二五”规划目标基本完成，“十三五”规划实施开局良好。

一、经济实力大幅提升。我们认真贯彻落实中央宏观调控政策，出台一系列激发活力、促进发展的政策措施，有效应对复杂严峻的经济形势，实体经济不断壮大，财税金融运行平稳，人民生活持续改善。据初步测算，2016年全市生产总值978亿元，比上年增长7%左右，年均增长9%；一般公共预算收入46.2亿元，与上年持平，年均增长11.6%；规模以上工业企业达到876家，拥有各类市场主体91229户；社会消费品零售总额494.9亿元，年均增长12.3%；五年累计完成固定资产投资1653亿元，年均增长21.3%；外贸出口总额27.6亿美元，年均增长0.4%，外商实际投资增幅居全省首位；城乡居民人均可支配收入1.8万元，年均增长10.5%。

二、产业升级步伐加快。我们坚持创新驱动发展，调整优化产业结构，构建现代产业体系，提升产业发展质量。我市被认定为国家知识产权试点城市，专利申请量和授权量均居粤东西北各市第2位，获得中国专利金奖、省科技一等奖各1项；工业技术改造投入增幅连续两年居全省各市首位，高新技术企业达到53家；凤泉湖高新区更名、扩大范围获得省批准，“市区镇企”四级创新服务平台建设成效明显，质检机构、专业镇支撑能力增强，主导或参与国家行业地方标准制修订99项，建成省级工程技术研发中心39家，省级以上企业技术中心42家，拥有省级以上名牌名标202项。粮食种植面积保持稳定，单丛茶、蔬菜、水果、畜牧、水产养殖等特色农业加快发展，拥有市级以上农业龙头企业41家、农民专业合作社838家。国家出口陶瓷质量安全示范区和新型工业化产业示范基地成功落地，八大特色产业集群不断壮大，新能源、新材料、生物医药等新兴产业加速发展，民营骨干企业示范引领作用增强，上市公司增至7家，挂牌企业45家，三环集团进入省民营企业100强。旅游经济长足发展，我市成为省旅游综合改革示范市，新增国家4A级旅游景区3处、全国重点文物保护单位1处，古城文化旅游特色区建设全面启动，预计2016年全市接待海内外游客1178.7万人次，实现旅游收入181.3亿元。现代服务业加快发展，“互联网+”扎实推进，我市入围全国电商百佳城市并名列第50位。

三、城市格局明显优化。我们紧扣省振兴粤东西北三大抓手，谋划推进“一中心三片区”建设，大力实施“八网”行动计划，基础建设突飞猛进，交通体系逐步完善，发展基础不断夯实。厦深高速铁路、潮惠高速公路建成通车，潮州港扩建货运码头试运行，广梅汕铁路增建二线及厦深联络线、梅汕客专、宁莞高速潮州段和大潮高速先行性工程开工建设，一批国省道改造提升，外环西路、绿榕西路、护堤路项目取得突破性进展，新改建县乡公路182公里、通村公路1155公里，“成型成网”交通体系初见成效。潮安内洋南总干排涝整治、饶平海堤达标加固一期等工程基本完成，引韩济饶供水工程启动实施，水利基础建设有效推进。燃气供应“一张网”、供电网建设改造进展顺利，光网、4G网络等建设力度加大，城市功能逐步完善。在全省地级市中率先编制全域规划，被列为国家和省新型城镇化综合试点市，城镇化率达到63.8%。韩东新城规划建设稳步推进，51个重点项目布点实施，恒大城、东方国际茶都等一批项目进展顺利，潮州大桥、如意大桥竣工在即，凤城公园建成开放。部分县区行政区划调整顺利完成，潮安实现撤县设区，饶平县城得到提升，高铁新城规划建设加快推进，城市呈多核化发展。园区支撑作用更加明显，中山（潮州）产业转移工业园“一区六园”格局形成。凤泉湖高新区动工超亿元项目71个，建成8个；闽粤经济合作区已动工项目18个，投产5个；东山湖现代产业园基础设施建设基本完成，已动工项目6个，投产项目2个。绿色发展示范区规划编制完成，“一镇一特”发展态势基本呈现。

四、生态环境保护有力。我们致力于改善环境，注重生态建设，深化社会治理，城市形象大幅提升。治脏、治水、治气“三治”工作持续开展，主要污染物排放总量有效控制，韩江综合整治成效突出，海洋生态得到较好保护。新一轮绿化潮州大行动深入开展，新建森林公园31个、湿地公园3个，森林覆盖率达到62.93%。违法用地、违法建设受到严厉打击，矿产资源管理工作不断强化，处置已供未用土地110宗306.7公顷，整治“两违”用地531宗1631.3亩。一批污水处理设施和潮安区垃圾焚烧发电项目加紧推进，第二污水处理厂开始试运行，潮安区被列为省“镇村污水处理项目”建设示范区，“户收集、村集中、镇转运、县处理”的垃圾收运处理模式基本建立。节能降耗工程常抓不懈，能耗约束性指标完成省下达的计划。

五、改革开放纵深拓展。我们坚持全面深化改革，不断完善科学发展的体制机制，构建高水平的开放型经济新格局。简政放权深入开展，对相关县区和经济开发区下放市级管理权限193项，实行2、3、4号章，行政效率大幅提高。营改增试点、商事制度等改革稳步推进，“土地一张图”、“燃气一张网”、“公交一张网”等取得突破，黄正村整村改造试点和土地承包经营权确权登记、“三资”交易平台建设等农村综合改革卓有成效，省级新农村示范片在全省率先实现县区全覆盖。对外交流合作持续深化，海丝、闽粤、中潮“三大合作”更加紧密，与厦门市、美国旧金山市、蒙特利公园市等建立友城关系，驻泰国经贸代表处正式挂牌运作，第六届粤东侨博会成功举办。汕潮揭同城化持续推进，与中山市“六个对接”深入开展，中山市累计投入帮扶资金8.7亿元，帮助引进投资额超过2000万元的产业项目272个。进出口贸易平稳发展，利用外资质量和水平有所提升，开放型经济更加活跃。

六、社会事业全面进步。我们把保障和改善民生作为社会建设的重点，大力发展各项社会事业，狠抓文明创建，全力办好每年十件民生实事，努力提高群众的获得感和幸福感。五年来，民生领域累计投入409亿元，占同期一般公共预算支出的70%以上。就业创业形势稳定，历年城镇登记失业率均控制在3%以内。城乡居民养老保险和医疗保险基本实现全覆盖，社会保障体系更加健全。人才工作积极开展，设立博士后科研工作站、创新实践基地10个，市人才驿站成为首批省市共建人才驿站。解困工程扎实有效，建成城镇各类保障性住房7489套，改造农村贫困户危房20611户，第一、二轮扶贫开发“双到”工作均圆满完成，163个贫困村10081户贫困户稳定脱贫，新时期精准扶贫精准脱贫三年攻坚全面启动。各类教育均衡协调发展，我市成为粤东首个省“教育强市”，“传承韩愈文脉、人人尊师重学”社会氛围日益浓厚。“卫生强市”启动创建，医疗服务水平稳步提升，登革热疫情有效防控，市中心医院成为全市首家三甲医院，人口计生工作进一步加强。文化惠民活动深入实施，各级公共文体设施场地全部免费开放，竞技体育取得新成效。非遗保护传承力度持续加大，市文化馆被评为国家一级馆。“韩愈教育发展基金会”、“潮州文化公益基金会”、“绿色生态发展基金会”创立运作。“平安潮州”建设扎实推进，盗抢、交通等违法犯罪受到强力打击，“飓风2016”成效明显，社会保持和谐稳定。法律援助更加有力，1015个村（社区）实现法律顾问全覆盖。安全生产责任制全面落实，食品药品监管体制日益健全，突发事件应急处置能力不断增强，五年来没有发生重特大生产安全事故和食品药品安全事件。文明创建活动持续开展，荣膺省双拥模范城市“八连冠”,“创业、精致、感恩、包容”成为全市共识，“海丝文化重镇、潮人精神家园”城市形象更加鲜明，创业拼搏的“红头船”精神和追求精致的“一根牛绳”精神深入人心。与此同时，人事、外事侨务、港澳、审计、统计、法制、司法、贸促、物价、粮食、打私、打假、人防、气象、防震、地方志、档案、对台、拥军优抚、民兵预备役、老龄、民族宗教、妇女儿童等工作取得新成绩。

在推动经济社会发展的同时，我们切实加强政府自身建设，全面落实党风廉政建设责任制，积极开展党的群众路线教育实践活动、“三严三实”专题教育和“两学一做”学习教育，落实好省委第九巡视组巡视整改工作，严肃查处各类违法违纪案件。坚持依法行政，自觉接受人大、政协监督，五年来累计办理人大代表建议192件、政协提案383件。

2016年是“十三五”的开局之年，面对经济下行压力，市政府围绕市委的总体部署，坚持多措并举，推动创新发展。一是启动“八网+产业”建设。在抓好年度投资163.6亿元的78个重点项目建设的基础上，编制三年行动计划，启动实施“八网+产业”重点项目建设，全年动工项目99个，累计完成投资86.6亿元。二是激发双创发展活力。出台一系列政策措施，培育扶持一批根植性强的本土民营企业做大做强，引导更多市场主体创业创新，全年培育小升规工业企业57家，建成首家科技企业孵化器，市众创空间被认定为国家级众创空间，7个专业镇上榜省专业镇创新指数，上榜数居粤东各市首位。成立潮州市“互联网+”研究院，凤泉湖“互联网+制造”小镇被列入省培育小镇，闽粤经济合作区成为2016粤东西北地区招商引资示范平台。三是加快产业园区发展。全力推进凤泉湖高新区开发建设，创造了“凤泉湖速度”，缔造了园区发展的“凤泉湖模式”，首期4500亩土地已全部供地，二、三期建设全面启动。中山（潮州）产业转移工业园、中山-潮州对口帮扶工作在省考核中均获得优秀等次。四是强化发展要素支撑。搭建“1+8+2”融资平台，向政策性银行上报融资项目47个，融资金额117.6亿元。推行政府和社会资本合作模式，有2个项目与社会资本成功合作。五是深化重点领域改革。大力推进供给侧结构性改革，落实40项重大改革措施。设立新的市政务服务中心，推行“一门式一网式”政务改革，实现100个部门1189项审批事项集中统一办理。开设政务服务“绿色通道”，63个服务项目审批时间压缩近一半。筹划设立新的市代建局，服务和保障政府投资重点项目建设。开展市级“大交通”管理体制和公交客运行业改革，粤运首批150台纯电动公交车陆续投入运营。六是维护社会和谐稳定。加强社会治安综合治理，全面开展城乡风险点、危险源排查整治行动，促进社会安全稳定。深化“文明镇街、文明村居”创建，建成覆盖各村居的乡贤咨询委员会。推进镇街环境综合整治，着力打造“一街、一路、一河、一厕”“四个一”工程，启动“三项帮扶、五项整治”试点工作，开展道路交通秩序百日整治大行动，不断改善城乡发展环境。

各位代表，回顾五年来的实践，我们深刻体会到，向着后发赶超、凤凰腾飞的目标迈进，必须坚持明晰发展定位，塑造发展新形象；必须坚持转方式调结构，引领发展新常态；必须坚持创新驱动发展，注入发展新动能；必须坚持深化改革扩大开放，构建发展新体制；必须坚持文明创建综合整治，营造发展新环境；必须坚持民生为重，共享发展新成果。

各位代表，过去五年改革发展的成绩来之不易，这是省委、省政府和市委正确领导的结果，是市人大、市政协有效监督和大力支持，以及驻潮部队、武警官兵、上级驻潮单位和社会各界鼎力相助的结果，更是全市广大干部群众辛勤努力、奋力拼搏的结果。在此，我代表市人民政府表示衷心感谢并致以崇高敬意!

在肯定成绩的同时，我们也清醒地看到，当前我市经济社会发展中还存在一些突出问题。主要是：经济底子较薄，产业层次不高，骨干企业不多，内外需求不足，经济面临较大下行压力，财政收支矛盾突出；基础设施不完善，投资环境亟待提升，补短板工作任重道远；协调各方利益、维护社会稳定压力加大，公共安全隐患不容忽视，生态文明建设还需努力，民生保障水平尚有差距；政府职能转变不快，行政效能有待提高，作风建设仍需加强。对此，我们一定高度重视并认真解决。

今后五年的奋斗目标和主要任务

未来五年，是潮州决战决胜，奋起直追，与全国同步建成小康社会的总攻时期。我们既面临经济压力、矛盾叠加的严峻挑战，也面临活力释放、动力增强的发展机遇。随着国家“一带一路”、创新驱动发展、供给侧结构性改革，以及省振兴粤东西北和对口帮扶等战略的深入实施，为我市加快发展注入新的动能。同时，我市“一中心三片区”发展格局的成型、各重大发展平台的引擎驱动、重大基础设施的配套完善，以及广大干部群众干事创业信心和决心的增强，包括旅外乡亲在内的投资者看好潮州、投资潮州，我们的发展将迎来强大“势能”。综上所述，当前潮州发展已到了从战略僵持转向全面进攻的关键节点。站在新起点，面对新期待，我们必须锁定目标、艰苦奋战，有力有为、见贤思齐，跑好“第一棒”，干出“第一棒”成绩，把美好蓝图变成现实!

做好今后五年政府工作的指导思想是：高举中国特色社会主义伟大旗帜，以邓小平理论、“三个代表”重要思想和科学发展观为指导，全面贯彻落实党的十八大、十八届中央历次全会和习近平总书记系列重要讲话精神，深入贯彻落实市第十四次党代会精神，以“凤凰腾飞”为总目标，按照“五位一体”总体布局和“四个全面”战略布局，认真落实“五大发展理念”，加快建设“一中心三片区”，着力打造新兴产业强市、交通强市、文化强市、旅游强市和教育强市，统筹推进经济社会建设，决战决胜，有力有为，实现两个“五年翻番”，与全国同步建成小康社会，把潮州建设成为经济更繁荣、政治更清明、社会更和谐的美丽新凤城。根据市第十四次党代会的部署要求，今后五年经济社会发展预期目标为：全市生产总值年均增长11%以上，人均生产总值年均增长10.5%以上，一般公共预算收入年均增长14%以上，固定资产投资年均增长25%左右，社会消费品零售总额年均增长13%，外贸进出口总额年均增长6.5%，城镇化率达到68%以上，城乡居民收入增长与经济增长基本同步。

为实现上述目标，必须下定决心、着眼长远，打造“五个强市”。即：依托现有优势，引进培育一批体量大、竞争力强的新产业集群、新产业门类、新产业模式，形成创新动力强劲、特色优势突出、平台支撑有力的新兴产业高地，打造新兴产业强市；加快高铁潮汕站扩容工程和潮州港建设，构建四通八达、无缝对接的综合交通网络，强化中心枢纽站和潮州港的辐射带动作用，实现交通功能与城市地位的同步提升，打造交通强市；大力整合资源，加强宣传推介，提升潮州文化海内外影响力，引进新兴文化形态，建设不失传统的现代潮州，推进文化与经济融合，繁荣文化事业，发展文化产业，打造文化强市；着眼于建设世界潮文化体验旅游目的地，迎合旅游发展新趋势，积极引进新业态，全面改善提升旅游发展环境，打造富有竞争力的旅游品牌，走出独具特色的旅游发展新路子，打造旅游强市；坚持教育优先发展，不断加大投入，调整优化布局，引进优质品牌，扶持民办教育，发展高职高等教育，提升教育综合实力，力争率先建成省教育现代化先进市，打造教育强市。按照这个要求，重点抓好五方面工作。

第一，实施“四梁八柱”工程，增强区域发展实力。坚持规划引领，突出“五个强市”新定位，加快“一中心三片区”建设，推动各片区协调发展。狠抓凤泉湖高新区、高铁新城、韩东新城、闽粤经济合作区等重大平台建设，科学布点产业项目，形成布局合理、产业集聚、特色鲜明的发展格局。搭建创新服务载体，鼓励支持新业态、新门类、新模式发展，培育形成一批新兴产业集群。加强扶持引导，大力培育一批根植性强的大型骨干企业，提升“中国瓷都”、“中国婚纱晚礼服”等区域品牌。

第二，实施“四面八方”工程，统筹推进各项事业。深入实施创新驱动战略，释放“双创”活力，激发经济社会发展潜能。以创建省教育现代化先进市为目标，优化办学门类、策略、资源、方法和手段，不断提升教育发展水平。以创建“健康潮州”为抓手，完善医疗卫生保障体系，促进人口均衡发展，加快各项社会事业发展。擦亮潮州文化品牌，注重精神文化引领，发展繁荣文化事业，打造不失传统的现代潮州。

第三，实施“四通八达”工程，着力优化发展环境。深入推进“八网”重点项目建设，谋划推进一批涵盖能源、交通、环保、防灾、水利、信息等体系的重点项目，不断提高基础设施现代化水平。加快国家新型城镇化综合试点市建设，大力推进新城规划建设和旧城改造，逐步完善城市功能。狠抓经济体制、投融资机制等重点领域改革攻坚，改善营商环境，抓好招商引资工作，扩大海丝、闽粤、中潮“三大合作”，增创开放型经济发展新优势。

第四，实施“四平八稳”工程，共同建设美好家园。聚焦城乡协调，全力办好每年十件民生实事，多渠道促进城乡居民增收。积极稳妥推进农村综合配套改革，打好新时期精准扶贫精准脱贫攻坚战。加强生态环境治理，加快绿色发展示范区建设，促进资源节约集约利用。坚持正本清源，整治各种社会乱象，提升基层治理能力，营造良好发展环境。抓好城乡环境卫生综合整治，完善城市管理体制，启动全国文明城市、全国卫生城市、国家环保模范城市、全国双拥模范城市、国家全域旅游示范区、国家森林城市“六城同创”工作，推动文明创建再上新水平。

第五，打造有力有为团队，建设人民满意政府。贯彻全面从严治党要求，始终把人民的期盼作为奋斗目标，认真履职，依法行政，主动担当，有力有为，努力在建设学习型政府、创新型政府、法治型政府、廉洁型政府、责任型政府、服务型政府上见实效，形成言必行、行必果和定了就干、干就干成、干就干好的施政新风，对人民负责，受人民监督，让人民满意。

我们坚信，经过五年的努力，潮州的发展一定会再上新台阶，城市将更加繁荣，生态将更加文明，社会将更加和谐，人民将更加幸福，一座不失传统的现代新凤城将以全新的姿态展翅翱翔！

2017年工作安排

2017年，是新一届政府的开局之年。综合考虑诸多因素，全市经济社会发展主要预期目标是：生产总值增长9%，一般公共预算收入增长11%，固定资产投资增长25%，外贸出口总额增长5%，社会消费品零售总额增长11%，居民消费价格指数涨幅控制在3%以内，城乡居民收入增长与经济增长基本同步，节能减排降碳约束性指标完成省下达的年度计划。

为实现上述目标，要认真贯彻落实中央、省有关会议和市第十四次党代会精神，以推进供给侧结构性改革为主线，以实施“百团大战”为引领，以建设国家新型城镇化综合试点市为抓手，集中优势力量，聚焦关键问题，增加有效投资，激发需求动力，深化创新驱动，推动产业转型升级，确保经济稳定增长，以优异的成绩迎接党的十九大胜利召开。

一、突出项目带动，强化发展支撑

坚持发展项目化，强化“项目为王、动工为先”理念，及早抓好年度投资164.65亿元的92个重点项目建设，继续推进“八网+产业”行动计划，着力补短板、强基础、蓄后劲。

抓实产业项目。坚持“实业兴市”，筑牢重大发展平台。抓住“凤泉湖速度”这一主核，瞄准“镇园一体化”的主攻方向，同步推进一期项目落地投产和二期用地征收及开发，全面完成中炬科技园、通用厂房、中山大道、科技孵化中心等项目建设，力促光明乳业、荣信系列项目、明园新型金属质感材料等产业项目加快进度；无缝对接汕潮揭临港空铁经济合作区规划，加快高铁新城核心区开发，推进莱芙生产基地、普洛斯物流园、佛手果加工基地等产业项目建设，启动东山湖现代产业园二三期征地工作，完成桑浦山功能定位和详细规划，大力发展梅林湖旅游商住项目；注重先后缓急，分步建设韩东新城各组团，完成意东片、北溪片等区域综合征地，抓好韩江新城半岛市民广场、桥东特色商业街、潮商城等项目建设，培育改造“潮创街”业态，推进韩东新城开发建设；深化与福建漳州、台湾的交流对接，推动区县合一，完成柘林、西澳等产业园区配套建设，加快实施LNG储配站、怡华油品仓储、粤东天然气接收站等项目，做大冻肉进口业务，规划形成装配式建筑生产基地，促进闽粤经济合作区发展；立足资源优势，规划建设茂芝红色经济区、汤溪湖旅游休闲小镇、赤凤森林古镇、文祠花果山度假区，探索发展农庄经济，推进绿色发展示范区建设。

抓实交通项目。推进实施年度总投资95.8亿元的61个交通重点项目，着重加快广梅汕铁路增建二线及厦深联络线、梅汕客运专线和动车运用所项目建设，争取潮汕站扩容工程动工，实现饶平站始发运营。抓好大潮高速、潮汕环线高速、汕汾高速连接凤泉湖新建工程等项目建设，做好宁莞高速与沈海高速连接线前期工作，确保宁莞高速建成通车。加速潮州港公用航道一期工程建设，实现潮州港扩建货运码头建成投入运营，做好潮州港公共锚地选划，完成潮州港亚太公司资产重组。推动潮州东大道和一批省道改扩建工程建设，启动潮汕大桥、凤凰大桥及连接线工程、北溪大桥建设，加快实施外环西路、护堤路潮安段、饶平沿海快速公路等项目，实现潮州大桥、如意大桥、如意路等通车，新改建县乡公路15公里、通村公路150公里，努力构筑外通内联、便捷通达的立体交通网络。

抓实水利项目。围绕“生态水乡网”建设计划，全面推进全市246宗水利项目建设。抓好韩东新城防洪、北堤、南堤（首段）、北溪和潮安西山溪等综合整治工作；加快引韩济饶供水工程、粤东灌区续建配套与节水改造工程、饶平海堤围达标加固二期工程和潮安内洋西总干排涝工程等建设；抓好中小河流综合整治及水系连通工程、农业高效节水项目和涝区整治等农村基础设施建设，不断提高水利保障能力。

抓实民生项目。着眼于提升人居环境和改善民生，提升市图书馆新馆和市档案馆，力争有30%的村居综合性文化服务中心建成。加快“博物馆之城”创建，全市博物馆总量达到30座。实施特殊教育学校工程，启动市高等职业技术学院建设，努力引进名校在潮办学。改造提升公立骨干医院办医水平，引进社会力量办综合三甲医院。全面完成径南和东山保障房建设，改造充实市城区肉菜市场。规划实施城区有偿停车体系，动工建设西门停车场及配套设施。启动一半以上乡镇建设市民亲水平台，推进凤城文化广场、凤城水乡生态示范区、红山森林公园、滨江休闲公园等项目建设，抓好城市主干道绿化和景观提升。

二、加快结构调整，促进产业提升

以供给侧结构性改革为主线，以创新驱动发展为引领，推进大众创业、万众创新，做大实体经济，培育壮大新动能，不断完善现代产业体系。

深化供给侧结构性改革。全面做好“三去一降一补”，推动供给侧结构性改革取得实质性进展。抓好“僵尸企业”出清重组，加快淘汰落后产能和化解过剩产能。加大棚户区改造力度，发展住房租赁市场，有序推动去库存，促进房地产健康发展。着力控制金融杠杆风险，优化金融生态环境，抓好农信社达标升级和改制为农商行工作。突出做好补短板和降成本工作，激发经济发展潜力。推进农业供给侧结构性改革，转变农业生产方式，带动农民增收。稳定粮食生产，保障粮食有效供给。支持设施农业项目发展，建成生态茶园1万亩以上。推进省级新农村连片示范工程建设，累计建成高标准农田22万亩以上。大力发展农产品深加工和流通服务业，拓展农村电商，促进农村一二三产业融合发展。积极推进畜禽养殖标准化示范创建，加快实施西澳岛生态修复工程，建设潮安北部万亩淡水养殖示范区。

坚持创新驱动发展。健全以企业为主体的技术创新体系。加快推进企业研发中心、科技企业孵化器、新型研发机构、产业技术联盟建设，建好国家级知识产权快速维权中心，促进产学研用贯通，推进产业链、创新链融合，建设创新发展示范镇5个以上，争取高新技术企业达到60家以上。聚焦特色优势产业，开展制造业升级行动，实施新一轮企业技术改造，推动传统优势产业向中高端跃升。深入实施质量强市战略，培育公共服务示范平台和创新产业化示范基地，建好创新驱动先行区，举办首届“市长杯”工业设计大赛，推动陶瓷、服装等行业实施自动化、智能化改造，促进传统优势产业与高科技嫁接、与设计联姻、与品牌联动。制定更加灵活的人才政策，大力培养引进创新人才。

培育壮大实体经济。实施产业龙头促进计划，打造根植于本土的“四梁八柱”骨干企业50家以上。加大对实体经济的支持力度，落实涉企收费清单制度，千方百计降低企业生产经营成本。完善“企业直通车”、“绿色通道”、“一企一策”等服务制度，继续开展民营骨干企业走访活动，帮助企业解决制约发展的问题。在韩东新城划设总部经济示范区，引导和扶持骨干企业集聚发展。加强直接融资服务，力争新增上市企业2家、挂牌企业6家。加大中小企业培育扶持力度，推动企业上规模，培育小升规工业企业68家。

推进新兴产业发展。加强对产业发展的规划引导，实施新兴产业行动计划，积极开展行业招商、产业招商，完善政策配套服务，集中力量发展工业陶瓷、生物医药和大健康、新能源、新材料（装配式建筑材料）等新兴产业，培育发展3-5个产值超过50亿元的新兴产业。推动互联网经济创新发展，建好凤泉湖“互联网+制造”小镇，加强大型物流园区、集散地和分拣中心建设，支持第三方物流企业融入生产企业供应链管理，鼓励发展快递业。大力发展软件和信息服务、科技服务、创意设计、服务外包等产业，壮大移动互联网、绿色、信息等新兴消费产业。

做大做强第三产业。发挥消费对增长的基础作用，积极发展现代服务业，引领和促进消费。强化供给创新，推动服务业发展与消费需求高水平对接。启动建设潮州美食城，规划设置陶瓷、不锈钢、茶叶、水产、二手车、农产品批发、家居装饰材料等专业市场，加快东方国际茶都、凤凰广场、财富中心等高端商贸综合体建设。积极发展文化创意产业，激活文化消费。以创建全域旅游为目标，加快古城文化旅游特色区建设，着重实施9个古城公共基础设施项目，引进大企业投建运营潮州古城，抓好旧府衙片区改造，开工建设文旅小镇、韩江沿堤慢道亲水走廊，打造笔架山潮州窑考古遗址公园，完善旅游厕所、停车场等设施建设，策划推介精品线路，举办潮府庙会、潮州美食节、潮州大锣鼓大赛、世界非遗论坛、文化艺术节、潮州节等系列文化旅游民俗活动，促进旅游产业发展。

三、抓好四项治理，优化发展环境

坚持问题导向，直面存在矛盾，集中力量，破解难题，整治经济社会发展弊病，优化城乡发展环境。

围绕建设法治化国际化营商环境，加强经济治理。整顿市场经济秩序，治理“假冒伪劣偷漏骗”，营造良好信用环境。深入推进镇村“三项帮扶、五项整治”，逐步解决农村产业发展、土地管理等方面存在问题，夯实基层经济基础。以“零容忍”态度严厉打击违法用地、违法建设、违法经营、违法居住、违法排放等现象，突出开展小产权房整治行动。鼓励专业镇发展提升，引导镇村企业集聚发展，实现土地集约、污染可控、管理规范。完善政府性债务管理和偿还机制，防范政府债务风险。依法加强镇街经济统计管理，建立经济运行考评制度，科学引导镇域经济发展。

围绕建设国家新型城镇化综合试点市，加强城乡治理。紧扣“一中心三片区”总体布局，创新城镇化发展模式，推进城乡基础设施和公共服务一体化，增强城乡综合承载能力，有序推进农业转移人口市民化，构筑新型城镇化空间局面，彰显潮文化特色和生态风貌。深入推进镇街文明创建活动，扩大创优覆盖面。实施农村环境连片综合治理，持续开展“治脏、治水、治气”专项行动，动工治理城乡污水河，巩固镇街“四个一”打造成效，基本完成“拆四旧”工作，落实长效管理机制，打造“三治”升级版。健全村务公开等制度，鼓励和支持乡贤咨询委员会、行业协会等参与社会治理，推进社会治理精细化。实施市城区综合配套改革，力争在整合行政资源、促进权责利统一、完善大执法体制等方面取得突破。

围绕建设“潮人生态家园”，加强生态治理。编制实施生态功能区规划，推进新一轮绿化潮州大行动，实施“绿道网”建设，新增镇级森林公园6个、湿地公园1个。加强生态创建，新增市级生态示范村12个、省级生态示范镇2个。严格水资源管理，启动韩江市区段饮用水源保护区范围调整工作，推进枫江流域污染整治，抓好河、溪、渠、沟的清污、清淤、清障。加快生活垃圾处理设施建设，完成“一县一场”建设任务，完善城乡垃圾收运处理体系。落实节能减排目标责任制，启动一批污水处理设施建设。严厉打击偷排污水、废气、废渣等环境违法行为，着力整治不锈钢行业抛光污染和陶瓷废弃物污染，不断优化生态环境。

围绕建设“平安潮州”，加强安全治理。创新完善立体化社会治安防控体系，严厉打击突出违法犯罪，推进安全文明小区和平安村居创建，全力打造粤东最平安城市。树立底线思维，抓好基层综治信访维稳工作，深化社会矛盾纠纷排查化解，维护社会和谐稳定。完善突发公共事件应急管理机制，增强灾害应急救援和防灾减灾能力。严格落实安全生产责任制，持续推进城市风险点、危险源排查整治和隐患排查治理工作，坚决遏制重特大生产安全事故发生。健全食品药品全流程监管体系，建设应用“智慧食药监”，建立市县镇三级统一信息网络，保障群众饮食用药安全。

四、深化改革开放，凝聚发展合力

汇集各方资源要素，营造创新创业环境，深化对外开放合作，汇聚发展正能量。

改善政务环境。深化行政审批制度改革，落实政府部门权责清单制度，实行行政审批标准化，拓展网上办事大厅，完善市县两级政务服务中心，推进市、县、镇、村四级“一门式一网式”政务服务全覆盖。规范和用好下放到园区的管理权限，实施投资项目全流程“保姆式”服务，推行“现场会”、“站谈会”等问题解决新模式，缩短项目入园落地时间。强化商事制度改革，推行工商注册便利化，探索全程电子化工商登记。开展市场准入负面清单制度改革试点，加强信用体系建设。深化国地税联合办税服务工作，探索推进办税业务由“一厅办”向“一窗办”转变。

推进改革攻坚。推进投融资体制改革，组建国有全资的城市发展投资建设集团，设立民营投资股份有限公司，强化产业发展支撑。深化财税管理体制改革，努力培植税源、挖掘潜力。抓好农村集体产权制度改革，规范引导农村土地承包经营权有序流转。推进燃气综合改革，加快城镇燃气管网、市域高压天然气管网建设，推动现有管道供气设施并购和整合提升，拓展天然气用气市场，尽快形成“燃气一张网”。抓好市级“大交通”管理体制改革后续工作，加快“公交一张网”建设。深化农电体制改革，推进新农网改造升级，抓好一批供电项目改造工程，提升配网供电可靠性。

提高开放水平。积极对接国家“一带一路”战略，全方位加强与“海丝”沿线国家的交流合作，深化与美国旧金山、泰国曼谷等的友城关系。依托闽粤经济合作区，全面拓展与福建、台湾的交流合作。加强与汕头、揭阳的对接，加快临港空铁经济合作区建设，扎实推进汕潮揭同城化发展。加快发展跨境电商，建设外贸综合服务平台，组织企业参加各类展销会，支持拓宽营销渠道，开拓多元化市场，促进外贸进出口稳定增长。

坚持合作发展。建立引资指导目录，灵活运用“精准招商”新模式，组织开展国内外招商推介活动，加强与珠三角地区产业共建，争取有更多大项目落户潮州。强化与广州港、厦门港的合作，提升港口带动和辐射能力，推动临港产业集聚发展。继续深化与中山市的对口合作，共同做好第二轮对口帮扶工作，推动两市专业镇协同创新，促进产业全链条跨区域融合发展。充分挖掘侨资侨力，深入实施“乡贤回归”工程，做好第二十届国际潮团联谊年会申办工作。

五、兜牢底线民生，推进发展共享

统筹社会事业发展，提升公共服务水平，保障和改善民生，不断提高群众的获得感。

做好就业保障工作。落实就业创业优惠政策，完善市创业孵化基地管理，建立初创企业及大学生创业创新平台，加强农村劳动力就业技能培训，抓好重点群体就业，新增城镇就业1.8万人，城镇登记失业率控制在3.5%以内。推进社保扩面征缴，完善社会保障体系，提高养老保险、医疗保险待遇水平。扎实抓好解困工程，推进新时期精准扶贫精准脱贫工作，落实城乡低保、农村五保供养标准，完成省下达的农村危房改造任务。

统筹社会事业发展。推进省教育现代化先进市创建工作，持续开展尊师重学系列活动，推动潮州大锣鼓进校园，加快发展职业教育、特殊教育、老年教育和学前教育，支持民办教育突破发展，逐步完善现代综合教育体系。继续深化医药卫生体制改革，全面推动卫生强市建设，强化重大疾病、传染病防控，试点公立医疗单位托管制度。实施优生健康惠民工程，促进人口均衡发展。广泛开展文化惠民活动，推进一批文体工程建设，完善城乡公共文化配套。成立凤凰城市联盟，加大中国瓷都、中国工艺美术之都形象宣传，培育更多工艺美术大师。继续做好民族宗教、外事侨务、港澳、广播电视、新闻出版、拥军优抚、国防动员、预备役、对台、人防、气象、防震、物价、档案、地方志、老龄、妇女儿童、残疾人、红十字等工作，促进各项事业全面发展。

认真办好民生实事。完善民生保障政策体系，加大民生投入，重点保障教育文化、医疗卫生、社会保障、扶贫济困等公共服务的支出。结合省的部署安排，聚焦群众普遍诉求，全力办好十件民生实事，切实兜牢底线民生。

六、加强政府建设，提高政务效能

以构建良好政治生态为目标，加强政府自身建设，打造有力有为团队，不断提高行政效能，为“凤凰腾飞”提供坚强保障。

建设法治政府。恪守依法行政基本要求，扎实抓好“七五”普法工作，促进自觉遵法学法守法用法。健全依法决策机制，加强合法性审查，推进行政决策科学化、民主化、法治化。坚持严格规范公正文明执法，深化行政执法体制改革。健全行政权力运行制约和监督体系，自觉接受人大、政协监督，广泛听取各方面的意见和建议，认真办理人大代表建议和政协提案。完善政府信息公开制度，重视发挥舆论监督、社会监督和审计监督的作用。

优化服务质效。全面正确履行政府职能，增强政府执行力、公信力。坚决破除思维定势，从墨守成规中走出来，从按部就班中走出来，从司空见惯中走出来，提高深化改革、推动发展、化解矛盾、维护稳定的能力和水平。保持有力有为的精神状态，不懈怠，不松劲，不回避矛盾，不推卸责任，营造肯干事、能干事、干成事的浓厚氛围。

强化督查落实。进一步完善大督查工作机制，整合督查力量，促进督查工作由单一推动向联合推动转变。建立重点督查项目库，设立电子台账，对督查项目实行统一编号、动态管理。建立专责监督执纪问责、容错纠错机制，强化政府绩效评估和行政问责，坚决纠正不作为、乱作为，坚决克服懒政、怠政，坚决惩处失职、渎职，着力解决缺位、越位、不到位的问题。

推进廉政建设。建立预防腐败清单制度，加强重大工程、重点项目和专项经费等领域的廉政风险防控，强化对公共资金、公共资源、国有资产的监管，严肃查处各类腐败案件。全面落实中央八项规定和党风廉政建设责任制，厉行勤俭节约，严格控制“三公”经费，把更多的财力用到惠民生、促发展上，力争做到干部清正、政府清廉、政治清明。

各位代表，新目标赋予新任务，新起点开启新航程。让我们紧密团结在以习近平同志为核心的党中央周围，在省委、省政府和市委的坚强领导下，不忘初心、勇立潮头，决战决胜、有力有为，打开新局面、创造新业绩，为实现“凤凰腾飞”美好愿景而努力奋斗！
